# The Effect of Psychological Intervention on Nutrient Status of Perioperative Patients with Lung Cancer

**Published:** 2018-04

**Authors:** Fen XUE, Fang HUANG

**Affiliations:** Special Ward 1, Shanghai Pulmonary Hospital, Shanghai 200082, P.R. China

**Keywords:** Psychological intervention, Perioperative period, Lung cancer, NRS2002, PG-SGA

## Abstract

**Background:**

To investigate the effect of psychological intervention on nutrient status of perioperative patients with lung cancer.

**Methods:**

Overall, 176 lung cancer patients admitted to Shanghai Pulmonary Hospital, Shanghai China from 2015 to 2016 were divided into control group (n=88) and study group (n=88). Conventional nursing strategy was adopted for patients in control group, while psychological nursing strategy was implemented for those in the study group, and the specific nursing efficiency was compared between the two groups. In addition to the assessment with Symptom Checklist 90 (SCL-90) and Quality of Life Questionnaire-Core 36 (QLQ-C30), patients were required to fulfill the evaluations with nutrition risk screening 2002 (NRS2002) and Patient-Generated Subjective Global Assessment (PG-SGA).

**Results:**

After nursing care, significant amelioration in scores of SCL-90 and QLQ-C30 of patients were seen in the study group (*P<*0.05). In the study group, the scores of NRS2002 and PG-SGA were all lower than those in the control group (*P<*0.001). Besides, PG-SGA score was also significantly correlated with the levels of serum total proteins, serum albumin, hemoglobin, reduction in diet and weight (*P<*0.01), while only reduction in diet and weight was obviously correlated with the score of NRS2002 (*P<*0.01). Moreover, a significant correlation was identified between the scores of PG-SGA and NRS2002 (*P<*0.01).

**Conclusion:**

Psychological intervention could effectively alleviate the psychological stress response and ameliorate the nutrient status of lung cancer patients in perioperative period, thereby reducing the negative feelings and increasing the life quality of patients.

## Introduction

Lung cancer is usually characterized with a very high mortality rate, and even in US, its mortality rate ranks 1st in all malignant tumors (1). Surgery is the major method for treatment of lung cancer (2, 3), including conventional thoracotomy and thoracoscopic surgery. With tremendous development, thoracoscopic surgery has gained huge success (4), but the prognoses of conventional thoracotomy and minimally invasive thoracoscopic surgery remain unsatisfactory. Some researchers (5, 6) have found that the nutrition status affects the prognosis of lung cancer patients to a certain degree, and put forward that appropriate nutrient support can shorten the length of stay in hospital and increase the life quality (7).

In this study, from the perspective of psychological nursing, we aimed to investigate the effect of psychological intervention on the nutrient status of lung cancer patients in perioperative period.

## Materials and Methods

### General materials

Through randomized sampling and grouping method, we selected 176 lung cancer patients who were admitted to the thoracic surgery department of Shanghai Pulmonary Hospital, China between May 2015 and May 2016, and divided them into two groups via randomized sampling and grouping method. In the control group (n=88), there were 50 males and 34 females with an average age of (51.37±4.53) yr old; while in the study group (n=88), there were 54 males and 38 females with an average age of (50.74±5.18) yr old. There were no statistically significant differences in the gender, age, smoking history and some other clinical features between two groups before the assessment of regular nursing strategy and psychological nursing strategy (Table 1).

**Table 1:** General characteristic materials of patients of the two groups

***General materials***	***Study group***	***Control group***	**P *value***
Age (yr)	50.74±5.18	51.37±4.53	0.087^*^
Gender (male/female)	50/38	54/34	0.171^**^
Smoking history (yes/no)	69/19	65/23	1.124^**^
Height (cm)	168±3.23	170±3.45	0.114^*^
Weight (kg)	57±5.76	56±6.23	0.212^*^
Education (primary school/junior school/high school/college or above)	26/32/21/9	27/33/20/8	0.095^**^
Pathological type (squamous carcinoma/adenocarcinoma	48/40	51/37	0.131^**^
TNM staging (I/II/III/IV)	29/45/14/0	31/44/13/0	0.231^**^
Lymphatic metastasis (yes/no)	53/35	51/37	0.203^**^

* *t* test;

** non-parameter test.

There were no statistically significant differences in comparisons of clinical data between the study group and the control group, including gender, age, height, weight, education, and staging of tumor node metastasis (TNM), etc., *P>*0.05

Inclusion criteria: a) Patients aged between 40 and 70 yr old with definite diagnosis of non-small cell lung cancer through pathological examination before surgery, conforming to the staging criteria of lung cancer in National Comprehensive Cancer Network (7th edition) and eligible for surgical treatment based on the general examination before surgery; b) Patients who were literate, so as to properly understand and independently answer the questions in questionnaires designed for this study; d) patients who had not received chemo-therapy or radiotherapy before surgery, and with no history of mental or psychological diseases.

The study was approved by the Ethic Committee of Shanghai Pulmonary Hospital. The patients were informed of the content and objective of this study and signed the written informed consent.

### Assessment

Ever since the 2nd day after admission to one day after surgery, conventional nursing strategy and psychological nursing strategy were implemented for patients in the control group and the study group, respectively, and patients were required to fulfill the assessments of Symptom Checklist 90 (SCL-90) (8) and Quality of Life Questionnaire-Core 36 (QLQ-C30) (9). Before assessment, nurses should explain the indexes of scale to patients, and patients were required to fill the questionnaires of SCL-90 and QLQ-C30 independently. Moreover, nutrition risk screening 2002 (NRS2002) (10) and Patient-Generated Subjective Global Assessment (PG-SGA) (11) were also conducted for 88 patients after surgery of lung cancer in each group. With the results of assessment, we compared the variations in scores of each scale of patients in the two groups before and after surgery, and before and after the psychological intervention for patients in the study group; we compared the changes in nutrient status of patients in the study group before and after surgery and psychological intervention.

### Psychological intervention method

Selection criteria for intervention nurses: Nurses should possess the 5-year or more expertise in thoracic surgery department in 3A hospital, or be qualified as the charge nurse with working experience of 2 year or longer; nurses should hold the college degree or above and be excellent in negotiation and coordination. Before assessment, all nurses in the intervention group attended standardized training courses, where they would comprehend the content of psychological intervention and assessment methods of each scale The content of intervention is shown as follows: Firstly, nurses should earn the trust of patients through establishing good nurse-patient relationship; in addition, nurses should fully understand the psychological requirement of cancer patients, thereby precisely evaluating the psychological problems of patients for specific psychological treatment; after surgery, charge physicians were required to explain the disease condition to patients, including tumor staging, postoperative chemotherapy or radiotherapy, or prognosis, and encourage the patients to be confident in treatment. At the same time, cognitive treatment should also be carried out for reducing the incidence of negative feelings, including: preoperative psychological support, emotional and cognitive intervention, behavior consciousness and group intervention. Simultaneously, nurses should actively cooperate with the charge physicians for symptomatic treatment (e.g. three-level analgesia, oxygen inhalation) to realize the comprehensive intervention, thereby accelerating the recovery of patients.

### Statistical analysis

SPSS 22.0 (Chicago, IL, USA) was used for data processing. Chi-square test was performed for enumeration data, while for comparison of measurement data, non-parameter test and *t* test were adopted. After the normal distribution test, data conforming to the normal distribution were presented as mean ± standard deviation. *t* test was performed for analysis of the difference between the study group and control group; for those not conforming to the normal distribution, they were presented as the median, and non-parameter statistic method was adopted. Kolmogorov-Smirnov test was performed for intergroup comparison, and spearman correlation analysis was conducted for identifying the correlation. *P<*0.05 suggested that the difference had statistical significance with α=0.05 as inspection level.

## Results

### Results of SCL-90 assessment

Results of normal distribution test showed that the results of SCL-90 assessment did not conform to the normal distribution, and, thus, non-parameter Kolmogorov-Smirnov test was carried out. Assessment results showed that significant amelioration in the psychological status of lung cancer patients in perioperative period was seen in the study group. In the study group, comparisons of the total score, mean score, number of positive item, mean score of positive symptoms and the scores of other factors, except for the psychosis-associated factors, showed differences with statistical significance (*P<*0.001), and the ameliorations were superior to those in the control group (*P<*0.001; Table 2).

**Table 2:** Assessment of SCL-90 in two groups (n=88)

***Group***	***Study group***	***Control group***	**P *value***
Anxiety	2.0	1.5	<0.01^*^
Depression	2.0	1.5	<0.01^*^
Paranoid	2.0	1.5	<0.01^*^
Obsessive-compulsive disorder	2.5	1.5	<0.01^*^
Psychosis-related factors	2.0	2.0	0.34
Horror	1.5	1.0	<0.01^*^
Somatization	1.5	1.0	<0.01^*^
Hostility	2.5	2.0	<0.01^*^
Other	1.5	1.0	<0.01^*^
Mean score of positive symptoms	2.5	2.0	<0.01^*^
Number of positive items	71.5	55.0	<0.01^*^
Total mean score	1.5	1.0	<0.05^*^
Total score	235.5	172.0	<0.05^*^

* *P<*0.05, and the differences have statistical significance

### Assessment results of QLQ-C30 before and after psychological intervention

Results of normal distribution test showed that the results of QLQ-C30 assessment did not conform to the normal distribution, and, thus, non-parameter Kolmogorov-Smirnov test was carried out. Results showed that there were statistically significant differences in body function, role function, feeling function and general life quality between the study group and the control group (*P<*0.05), and ameliorations were also observed in tiredness, sleep disorders, pain and decrease in appetite in the study group (Table 3).

**Table 3:** Assessment results of QLQ-C30 in two groups (n=88)

***Item***	***Study group***	***Control group***	**P *value***
Body function	56.5	67.5	<0.05^*^
Role function	60.5	71.5	<0.05^*^
Emotion function	54.5	71.5	<0.01^*^
Cognitive function	69.5	73.5	0.21
Social function	52.5	55.5	0.45
Total life quality	53.5	68.5	<0.01^*^
Tiredness	42.5	47.5	<0.05^*^
Nausea and vomiting	13.5	15.5	0.27
Pain	37.5	46.5	<0.05^*^
Dyspnea	49.5	52.5	0.23
Insomnia	36.5	44.5	<0.05^*^
Anorexia	35.5	45.5	<0.05^*^
Constipation	17.5	21.5	0.18
Diarrhea	16.5	20.5	0.26
Economic burden	50.5	53.5	0.35

* *P<*0.05, and the differences have statistical significance

### Assessment results of NRS2002 and PG-SGA after psychological intervention (Table 4)

As shown in the results of NRS2002 evaluation, the nutrition risk of the study group was 19.32% (17/88), and that of the control group was 28.41% (25/88); while the results of PG-SGA indicated that among the patients, the incidence rate of (B+C) malnutrition in PG-SGA was 22.73% (20/88), while that of the control group was 30.68% (27/88). Scores of NRS2002 and PG-SGA in the study group were significantly lower than those in the control group with statistically significant difference (*P<*0.01), in which the incidence rate of malnutrition measured by PG-SGA in the two groups was higher than that by NRS2002, and the difference had statistical significance (*P<*0.01). Consistency check of NRS 2002 and PG-SGA showed kappa = 0.74 (*P<*0.01), suggesting that the results of PG-SGA and NRS2002 assessments were consistent.

**Table 4:** Assessment results of NRS2002 and PG-SGA

***Item***	***NRS2002 (n)***		***PG-SGA (n)***		
	≥3 points	< 3 points	A	B	C
Study group	17	71	68	14	6
Control group	25	63	61	17	10

Note: NRS2002 for nutrition risk screening, and PG-SGA for Patient-Generated Subjective Global Assessment

### Correlations between assessment results of NRS2002 and PG-SGA and different nutrient indexes in the study group

PG-SGA score was significantly correlated with the levels of serum total proteins, serum albumin, hemoglobin, reduction in diet and weight (*P<*0.001), while only reduction in diet and weight was obviously correlated with the score of NRS2002 (*P<*0.05; Table 5).

**Table 5:** Correlations between assessment results of NRS2002 and PG-SGA and different nutrient indexes in the study group

***Item***	***NRS2002***		***PG-SGA***	
	r	*P*	r	*P*
Age (yr)	−0.053	0.672	0.008	0.896
Height (cm)	−0.045	0.676	0.011	0.093
Weight (kg)	−0.188	0.121	−0.191	0.089
Total protein (g/l)	0.048	0.724	−0.497	0.003
Albumin (g/l)	−0.081	0.496	−0.521	0.000
Hemoglobin (g/l)	−0.179	0.124	−0.434	0.007
Reduction in diet (%)	0.812	0.000	0.684	0.000
Reduction in weight (%)	0.861	0.000	0.783	0.000

## Discussion

Despite the significant increase in survival rate of lung cancer patients after resection according to the previous studies (12, 13), surgical resection also gives rise to the psychological problems in many patients for postoperative complications, extended surgical duration of thoracic surgery, heavy trauma and postoperative pains. Some studies (14, 15) have discovered that psychological problems can promote the development and progression of cancer through immunological dysregulation. Moreover, psychological problems can also lead to an increase in mortality rate of cancer patients. Thus, comprehensive psychological nursing intervention is necessary for patients (16, 17). Some literatures (18–20) reported that the nutrient status of patients is closely correlated with the development, progression and prognosis of tumors, but the effect of psychological intervention on nutrient status of cancer patients has not yet been fully investigated. Hence, in this study, from the perspective of psychological nursing, we researched the effect of psychological nursing on nutrient status of lung cancer patients. In this study, through the assessments with SCL-90 and QLQ-C30, the positive effect of psychological nursing was ascertained. The results of this study showed that after nursing care, significant amelioration in scores of SCL-90 and QLQ-C30 of patients were seen in the study group, which was superior to the control group, and the difference had statistical significance (*P<*0.05); significant amelioration in the psychological status of lung cancer patients in perioperative period was seen in the study group, and comparisons of the total score, mean score, number of positive item, mean score of positive symptoms and the scores of other factors, except for the psychosis-associated factors, showed differences with statistical significance (*P<*0.001), which are coincident with some literatures (21–23).

Psychological intervention before and after surgeries for cancer patients is critical to reducing the anxiety and stress response of patients, and after psychological intervention, the tolerance of patients to pains will be enhanced. Furthermore, we detected the effect on nutrient status of lung cancer patients with assessments of NRS2002 and PG-SGA. NRS2002, with the operability and simplicity, excels in screening and monitoring the nutrient risks (24, 25), and can easily identify the patients with early-stage malnutrition, which is conducive to the prophylaxis of malnutrition. In US, PG-SGA is preferred in nutrition screening (25).

Due to the patients’ mental burden and refractory feature of the disease, the incidence rate of negative feelings in patients is far higher than that in the healthy population, and these feelings can significantly affect the nutrition and life quality of patients (26). In this study, we found that after psychological intervention, the nutrient status in the study group was significantly better than that in the control group. Furthermore, results indicated that PG-SGA score was significantly correlated with the levels of serum total proteins, serum albumin, hemoglobin, reduction in diet and weight (*P<*0.05), while only reduction in diet and weight was obviously correlated with the score of NRS2002 (*P<*0.05), and the differences had statistical significance. This study is also limited by the bias generated from the single center study. Thus, multicenter studies are expected to confirm further the effectiveness of psychological nursing care.

## Conclusion

Psychological intervention can effectively alleviate the psychological stress response, and ameliorate the nutrient status of lung cancer patients in perioperative period, thereby reducing the negative feelings, increasing the life quality of patients and benefiting the postoperative recovery of patients with a reduction of length of stay in hospital. Thus, psychological intervention deserves to be promoted in nursing care of cancer patients by medical professionals.

## Ethical considerations

Ethical issues (Including plagiarism, informed consent, misconduct, data fabrication and/or falsification, double publication and/or submission, redundancy, etc.) have been completely observed by the authors.
